# Transversus Abdominis Plane Block in the Emergency Department for Paracentesis: A Case Report

**DOI:** 10.7759/cureus.106342

**Published:** 2026-04-02

**Authors:** Mit Patel, Arqam Husain, Revelle Gappy, Andrew Park, Christopher R Clark

**Affiliations:** 1 Emergency Medicine, University of South Florida, Tampa, USA; 2 Emergency Medicine, Henry Ford Health System, Detroit, USA

**Keywords:** pain management, paracentesis, peritonitis, regional anesthesia, sbp, tap block, transversus abdominis plane block, ultrasound

## Abstract

Abdominal paracentesis is a key diagnostic and therapeutic intervention in patients with cirrhosis and ascites, yet it can be painful, especially in the presence of peritonitis. While local anesthetic infiltration is the conventional method for pain management, regional anesthesia techniques such as the ultrasound-guided transversus abdominis plane (TAP) block offer an effective, opioid-sparing alternative. We present the case of a 33-year-old woman with cirrhosis and suspected spontaneous bacterial peritonitis who underwent a large-volume paracentesis in the emergency department following a TAP block. The block provided effective somatic analgesia, improved procedural tolerance, and minimized opioid use. The case highlights the utility of TAP blocks as part of a multimodal pain management strategy for paracentesis in select emergency department patients. Broader implementation may enhance patient comfort, reduce the need for systemic analgesia, and provide valuable procedural training opportunities for emergency physicians.

## Introduction

Abdominal paracentesis is a key diagnostic and therapeutic intervention in patients with cirrhosis complicated by ascites. When peritonitis is suspected, a diagnostic paracentesis is a crucial but often painful intervention, particularly in patients with inflamed peritoneum or large-volume ascites. The American Association for the Study of Liver Diseases (AASLD) guidelines now recommend a diagnostic paracentesis upon admission in the diagnostic evaluation of a patient with ascites, which is likely to increase the number of paracenteses performed in the emergency department (ED) [1]. The conventional method of pain management for paracentesis is infiltration of a local anesthetic agent injected into the skin and soft tissue down to the peritoneum at the planned needle or catheter insertion site [2].

Ultrasound-guided regional nerve blocks have been increasingly utilized in the ED to provide effective regional analgesia. The transversus abdominis plane (TAP) block is a well-established regional anesthesia technique typically used for post-operative pain management in abdominal surgeries, with numerous studies supporting its efficacy [3]. The TAP block involves injecting a local anesthetic in the plane between the transversus abdominis and internal oblique muscles [4]. The interfascial plane contains the intercostal, subcostal, iliohypogastric, and ilioinguinal nerves that give sensation to the anterior and lateral abdominal wall [4]. Case studies have reported the use of TAP blocks in the ED for conditions such as appendicitis, rectus sheath hematomas, renal colic, and abdominal wall abscesses [5-8]. To our knowledge, there are no cases describing the efficacy of the ultrasound-guided TAP block in the ED as a part of a multimodal pain pathway for patients undergoing paracentesis with suspected peritonitis. We describe a case of a patient with cirrhosis and suspected peritonitis who successfully underwent a large-volume paracentesis in the ED using a TAP block, with subsequent improvement in pain control.

## Case presentation

**Figure 1:**
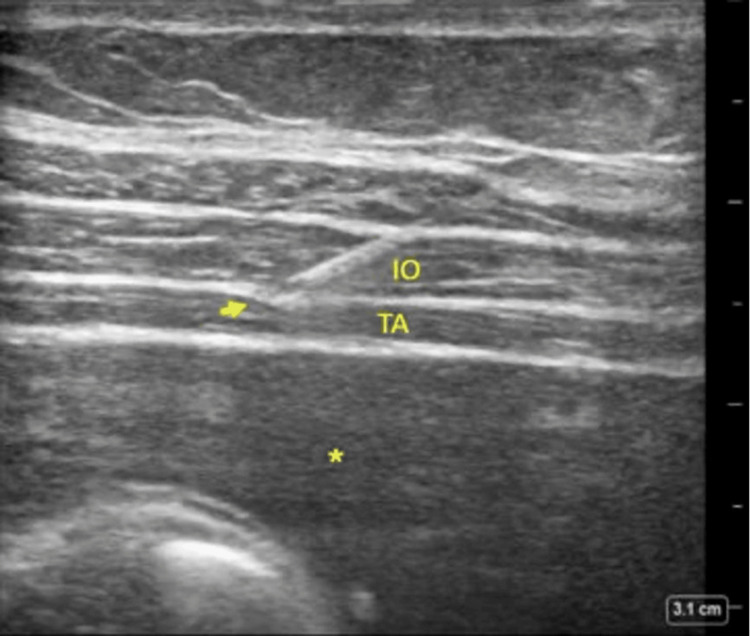
TAP Block Needle Position Needle tip (arrowhead) inserted in the transversus abdominis plane (TAP) between internal oblique (IO) and transversus abdominis (TA) muscle with visualized ascitic fluid (*)

**Figure 2:**
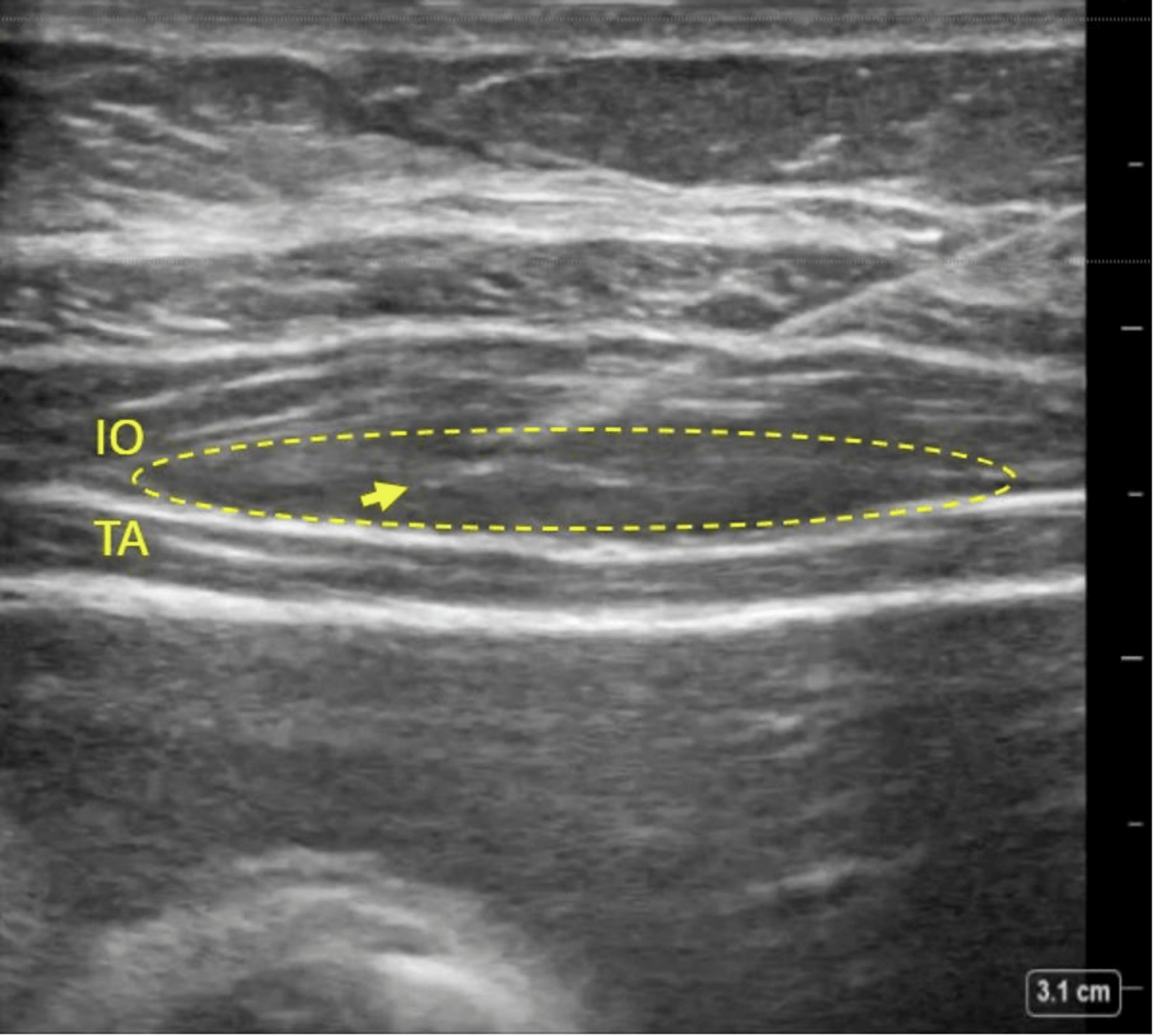
TAP Block Plane Separation Anesthetic spread (dashed line) along transversus abdominis plane (TAP) after bupivacaine injection. Needle tip (arrowhead) remains between internal oblique (IO) and transversus abdominus (TA) muscle groups.

A 33-year-old female with a history of cirrhosis presented to the ED with weight gain, abdominal pain and distension, fevers, and chills. She had a history of frequent admissions for decompensated alcoholic cirrhosis requiring recurrent paracenteses and recent band ligation of esophageal varices. ED vital signs were notable for tachycardia. Her clinical presentation raised concern for spontaneous bacterial peritonitis (SBP), and a paracentesis was indicated. Prior to performing the paracentesis, an ultrasound-guided TAP block was performed using bupivacaine in the right lower quadrant of the abdomen with the patient in the supine position. The area was prepped and draped in sterile fashion, and a high-frequency linear ultrasound probe was placed in the axial plane along the lateral abdominal wall to identify the fascial plane between the internal oblique and transversus abdominis muscles. Using an in-plane technique, a 20-gauge needle was advanced under direct visualization, and 30 milliliters of 0.25% bupivacaine without epinephrine was injected into the transversus abdominis plane at the intended procedural site (Figures 1-2).

Before the block, the patient rated her abdominal pain as 8/10 in severity. Forty-five minutes after the block, she endorsed numbness to the right hemi-abdomen with complete sensory loss to pinprick. She endorsed feeling pressure but denied pain during insertion of the paracentesis needle. At the patient’s request, additional local infiltration with 7 milliliters of 1% lidocaine without epinephrine was administered to complement the nerve block at the skin insertion site within the area covered by the TAP block, after which the patient endorsed complete anesthesia. The paracentesis yielded 2,200 milliliters of ascitic fluid and was tolerated without complication. Following the procedure, the patient reported improved pain for the remainder of her ED stay. Fluid studies ruled out SBP and ultimately the patient was admitted to the hospital and discharged after six days.

## Discussion

Ascites and associated cirrhosis-related complications, such as SBP, are commonly encountered in the ED. This case illustrates a novel application of the ultrasound-guided TAP block in the ED for procedural analgesia in patients undergoing paracentesis and concomitant pain control for peritonitis, particularly in the context of suspected SBP. Currently, local anesthetic infiltration is the standard practice for pain control during paracentesis [2]. However, studies have increasingly demonstrated the advantages and benefits of multimodal pain control in the acute ED setting [9]. While TAP blocks are commonly used in the operative setting and described in case reports for other ED indications such as renal colic and appendicitis, there is limited literature on their application for peritoneal fluid drainage and concomitant pain control for peritonitis [3,5,7,10]. This case demonstrates that emergency physicians can perform ultrasound-guided TAP blocks in the ED to achieve effective somatic analgesia prior to paracenteses and improve patient comfort and procedural tolerance. Furthermore, TAP blocks can help decrease reliance on systemic opioids and enhance procedural success, especially in patients with cirrhosis who may have contraindications to systemic sedatives or pain medications. In our case, despite the patient demonstrating pinprick anesthesia following the TAP block, she requested additional topical administration of lidocaine. The authors believe this request was driven more by procedural apprehension rather than inadequate analgesia, as she made the request a few minutes after confirming anesthesia and only after seeing the large paracentesis needle. Nonetheless, patient comfort and satisfaction must be acknowledged - especially during elective procedures. Following topical administration of local anesthetic at the insertion site, the patient experienced complete surgical anesthesia.

With the AASLD now recommending a diagnostic paracentesis upon hospital admission for patients with cirrhosis and ascites, ED-performed diagnostic paracenteses are likely to become more frequent - expanding the role of emergency physicians in ensuring patient comfort during painful procedures. Incorporating ultrasound-guided regional anesthesia into standard ED practice can be an invaluable tool in this regard. The TAP block is widely regarded as a relatively safe and simple regional anesthesia technique to perform. Incorporating a TAP block more frequently can help early learners obtain an adequate number of repetitions to develop transferable micro-skills that can later be applied to more technically challenging regional anesthesia procedures.

There are important limitations to consider. The anesthetic agent that is commonly used in TAP blocks is bupivacaine, which has an onset of action of five to 10 minutes, with the peak effect being approximately 30 to 45 minutes and the duration of action ranging between two and 10 hours [11,12]. In our case, the nerve block was allowed sufficient time to reach peak effect by waiting until the bupivacaine set in, prolonging what was already a time-intensive procedure in a busy ED. Additionally, unlike a routine paracentesis, safe performance of a regional anesthesia procedure requires placing the patient on a cardiac monitor and may also require post-procedural monitoring, depending on institutional protocols, due to the potential risk of local anesthetic systemic toxicity (LAST) from inadvertent intravascular injection. Furthermore, while bupivacaine is widely regarded as a safe and effective local anesthetic agent, there is a theoretical concern regarding its potential for drug-induced liver injury which has been raised in emerging case reports [13]. However, no consistent mechanism or causal relationship has been established, and most cases remain isolated and confounded by polypharmacy, comorbid liver disease, or other perioperative exposures. As bupivacaine is primarily metabolized by the liver, impaired hepatic function in cirrhotic patients may reduce clearance and increase the risk of systemic toxicity. Additionally, given its relatively higher cardiotoxic potential, alternative agents such as ropivacaine may be considered in this population. These limitations can add a layer of complexity, thereby limiting widespread adoption. Despite these limitations, this case supports the utility of TAP blocks in select ED patients undergoing paracentesis. It also suggests that TAP blocks are a valuable component of a multimodal pain control management approach in the ED, particularly in patients with significant procedural pain or peritoneal inflammation.

## Conclusions

Ultrasound-guided TAP blocks are a safe and effective adjunct for pain control in patients undergoing paracentesis in the ED. This case highlights the value of regional anesthesia as part of a multimodal pain strategy, especially in patients with severe ascites and concern for peritonitis. Future studies are needed to evaluate the efficacy and optimal protocols for broader implementation in ED practice.

## References

[1] Biggins SW, Angeli P, Garcia-Tsao G (2021). Diagnosis, evaluation, and management of ascites, spontaneous bacterial peritonitis and hepatorenal syndrome: 2021 practice guidance by the American Association for the Study of Liver Diseases. Hepatology.

[2] Koyfman A, Long B (2018). Peritoneal procedures. Roberts and Hedges’ Clinical Procedures in Emergency Medicine and Acute Care.

[3] Mavarez AC, Hendrix JM, Ahmed AA (2023). Transabdominal plane block. StatPearls.

[4] Tsai HC, Yoshida T, Chuang TY (2017). Transversus abdominis plane block: an updated review of anatomy and techniques. Biomed Res Int.

[5] Mahmoud S, Miraflor E, Martin D, Mantuani D, Luftig J, Nagdev AD (2019). Ultrasound-guided transverse abdominis plane block for ED appendicitis pain control. Am J Emerg Med.

[6] Stenberg RT, Wahi-Singh B, Wahi-Singh P, Hill A, Simon EL (2023). Transversus abdominis plane (TAP) block for pain management of rectus sheath hematoma in the emergency department (ED). Am J Emerg Med.

[7] Kadioglu E, Kaya M, Yildirim H (2020). Transversus abdominis plane block: a new method in renal colic pain management. Am J Emerg Med.

[8] Herring AA, Stone MB, Nagdev AD (2012). Ultrasound-guided abdominal wall nerve blocks in the ED. Am J Emerg Med.

[9] Nagpal AK, Gadkari C, Singh A, Pundkar A (2024). Optimizing pain management in emergency departments: a comprehensive review of current analgesic practices. Cureus.

[10] Kitaba S, Tsukada S, Takahashi K (2021). Transversus abdominis plane block for placement of a paracentesis catheter with failed Fontan physiology. Anaesth Pain Intensive Care.

[11] Beiranvand S, Moradkhani MR (2018). Bupivacaine versus liposomal bupivacaine for pain control. Drug Res (Stuttg).

[12] Kalu R, Boateng P, Carrier L, Garzon J, Tang A, Reickert C, Stefanou A (2021). Effect of preoperative versus postoperative use of transversus abdominis plane block with plain 0.25 % bupivacaine on postoperative opioid use: a retrospective study. BMC Anesthesiol.

[13] Chintamaneni P, Stevenson HL, Malik SM (2016). Bupivacaine drug-induced liver injury: a case series and brief review of the literature. J Clin Anesth.

